# Viscoplastic properties of laponite-CMC mixes

**DOI:** 10.1016/j.dib.2017.02.002

**Published:** 2017-02-14

**Authors:** Z. Tarhini, S. Jarny, A. Texier

**Affiliations:** Institut Pprime, Université de Poitiers, CNRS, ISAE-ENSMA, F-86962 Futuroscope-Chasseneuil, France

**Keywords:** Viscoplastic, Rheology, Laponite, CMC

## Abstract

In this dataset, 15 samples of laponite-CMC mixes were realized and their viscoplastic properties are determined. Rheological parameters are then expressed as a function of age and components concentrations.

**Specifications Table**

**Table:** 

Subject area	*Physics*
More specific subject area	*Viscoplastic properties*
Type of data	*Table, graph*
How data was acquired	*Rheometer (Gemini, Malvern)*
Data format	*Raw, analyzed*
Experimental factors	*15 samples of laponite-CMC were made with different concentrations of components (synthetic clay and polymer) and analyzed with a rheometer*
Experimental features	*Rheometrical tests are carried out and viscoplastic properties are linked to the concentration of components*
Data source location	*n/a*
Data accessibility	*Data is available in this paper*

**Value of the data**

• The data can be compared to other recipes to obtain transparent viscoplastic mixes for optical measurements such as PIV.
• The data can be used directly to make transparent viscoplastic model fluids with rheological controlled properties for civil engineering, agroalimentary, sediment transport, cosmetic field,…
• The data show effects of clay and polymer concentrations on viscoplastic parameters to reach the desired rheological properties.

## 1 Data

Rheological properties on 15 samples of laponite-CMC mixes are determined varying mass concentration of the components (laponite and carboxymethylcellulose) [1], [2]. Rheograms are established and a viscoplastic model (Herschel–Bulkley) is fitted. Model parameters are expressed as a function of time or components concentration.

## 2 Experimental design, materials and methods

### 2.1 Materials

Transparent mixes are prepared from a synthetic clay (laponite RD, Rockwood) and polymer (carboxymethylcellulose (CMC), Prolabo) [3]. For the polymer solution, CMC powder is poured gently to deionized water stirred with a magnetic agitator (C-100, Prolabo) at 600 rpm. The solution is then stirred for 1 h more at 600 rpm. For the laponite suspension, laponite powder is poured gently to deionized water stirred with a homogenizer (Ultraturrax T25, IKA) at 11,000 rpm. The suspension is then stirred for 15 min more at 11,000 rpm. The preparation of laponite suspension begins 15 min before the end of the stirring period of CMC solution. Then laponite suspension is poured on the CMC solution and the mixture is stirred for 1 h more with a magnetic agitator (C-100, Prolabo) at 1100 rpm. Mixtures are then put in hermetic flacons and let at rest before experiments at ambient room temperature (almost 20 °C). Each batch has a weight of 150 g and masses of laponite and CMC are calculated from this reference.

### 2.2 Rheometrical tests

Rheometrical tests are carried out with controlled stress rheometer (Gemini, Malvern) using plate-plate geometry of 4 cm diameter. Both surfaces are covered with sandpaper to prevent slippage effects. Each measurement is realized for a 1 mm gap and temperature is kept constant at 20 °C.

**Fig. 1:**
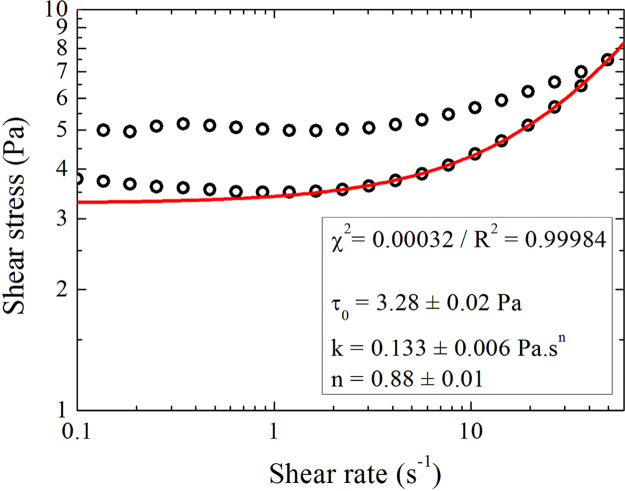
Fitted curve with Herschel–Bulkley model on sample 0.5% CMC+0.5% laponite for D+1.

The procedure begins with a pre-shearing phase to put samples in an initial state and to ensure reproducible results. It consists on applying a constant shear rate (10 s^−1^ during 120 s) following by a rest period (600 s). Flow curves measurements are then obtained by applying shear rate steps ranging from 0.1 to 50 s^−1^ in a logarithmic repartition, first by increasing values follow by decreasing values. Each shear rate step is applied for 40 s but only the last 10 s are averaged to obtain a measure point. Down flow curve is fitted with a Herschel–Bulkley model (Fig. 1) to determine yield stress $\left(\tau_{0}\right)$, consistency (k) and viscosity index (n):

$$\tau =\tau_{0}+k{\dot{\gamma }}^{n}$$

Measurements are realized on 15 samples for three different ages: D+1; D+7 and D+20 where D is the day of sample making. For each day, three measurements are made and determined Herschel–Bulkley parameters correspond to the average of these three measurements (Fig. 2, Fig. 3, Fig. 4, Fig. 5, Fig. 6, Fig. 7, Fig. 8, Fig. 9, Fig. 10, Fig. 11 and Table 1).

## Figures and Tables

**Fig. 2:**
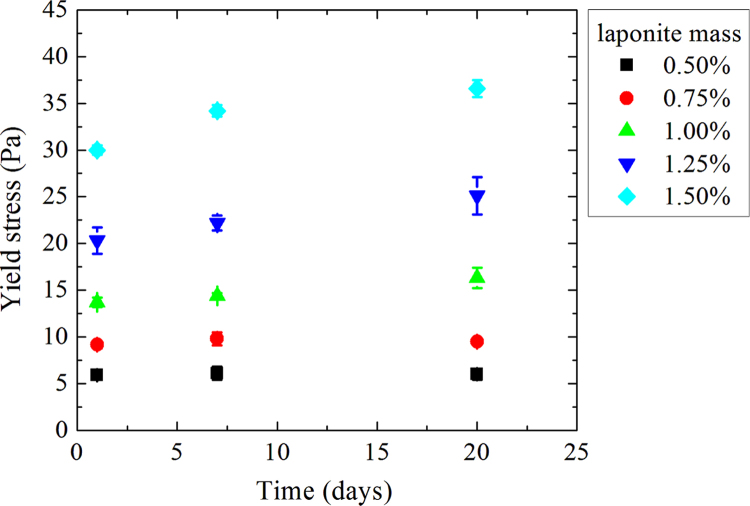
Yield stresses as a function of time for a CMC mass concentration of 0.75%.

**Fig. 3:**
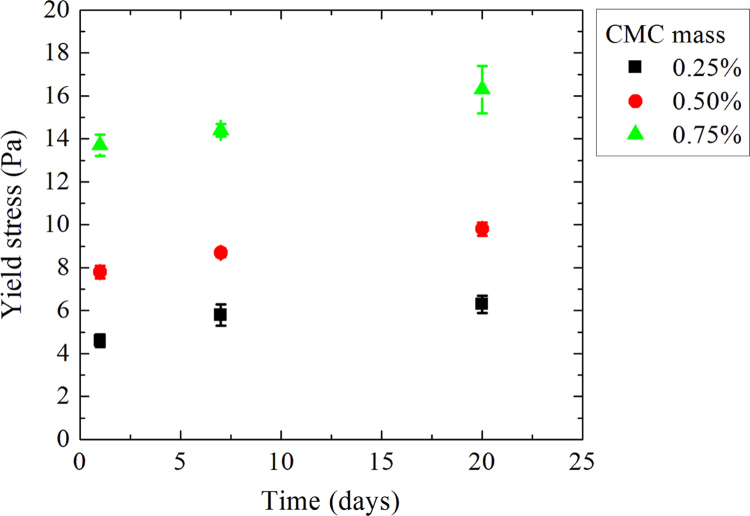
Yield stresses as a function of time for a laponite mass concentration of 1.0%.

**Fig. 4:**
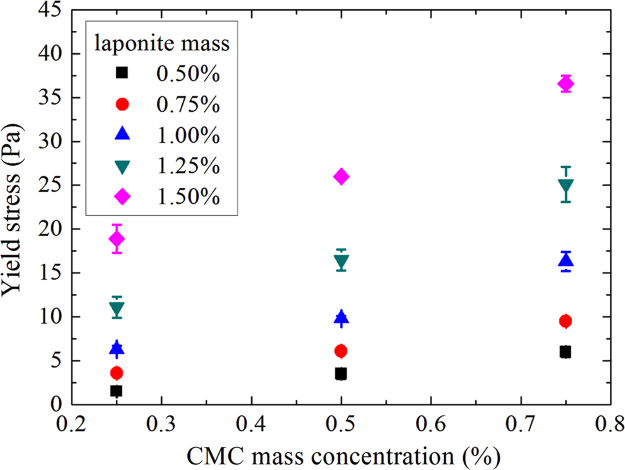
Yield stresses as a function of CMC mass concentration for D+20.

**Fig. 5:**
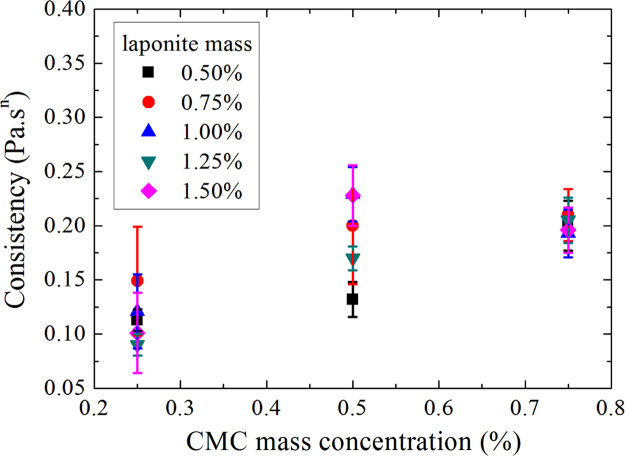
Consistencies as a function of CMC mass concentration for D+20.

**Fig. 6:**
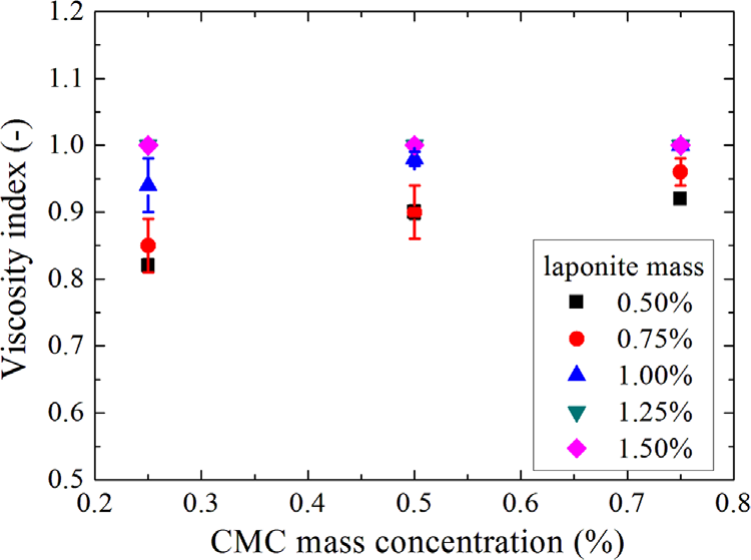
Viscosity indexes as a function of CMC mass concentration for D+20.

**Fig. 7:**
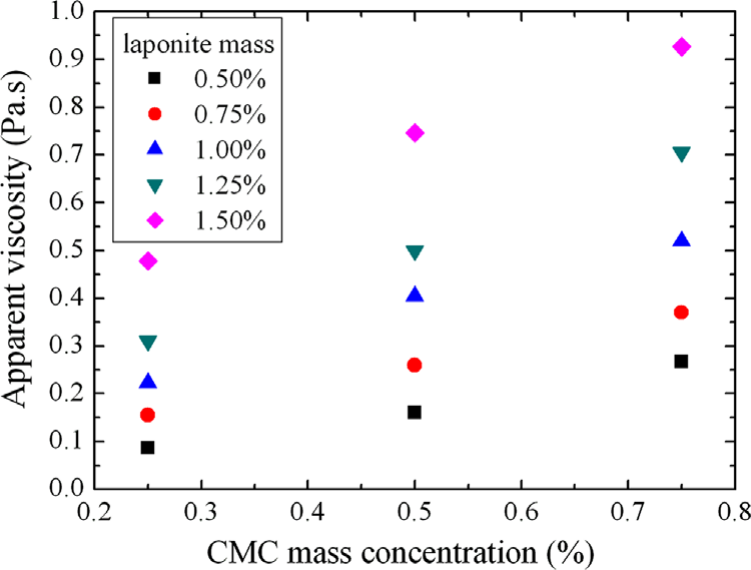
Apparent viscosity for $\overset{˙}{\gamma }=50\;s^{-1}$ as a function of CMC mass concentration for D+20.

**Fig. 8:**
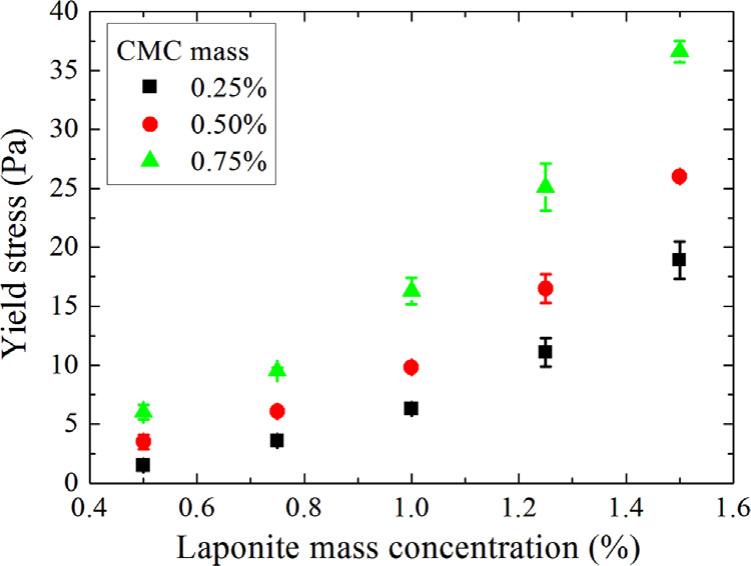
Yield stresses as a function of laponite mass concentration for D+20.

**Fig. 9:**
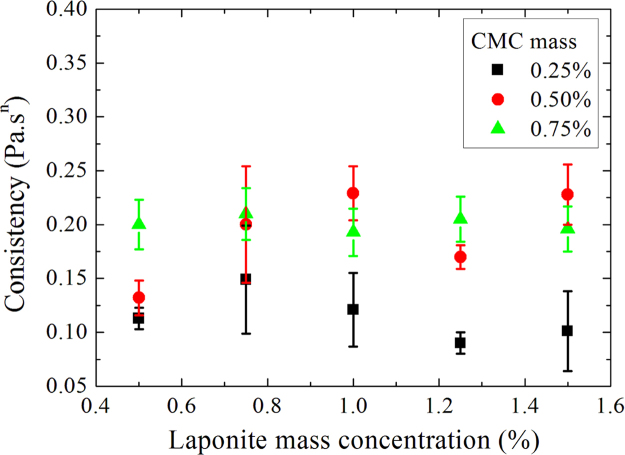
Consistencies as a function of laponite mass concentration for D+20.

**Fig. 10:**
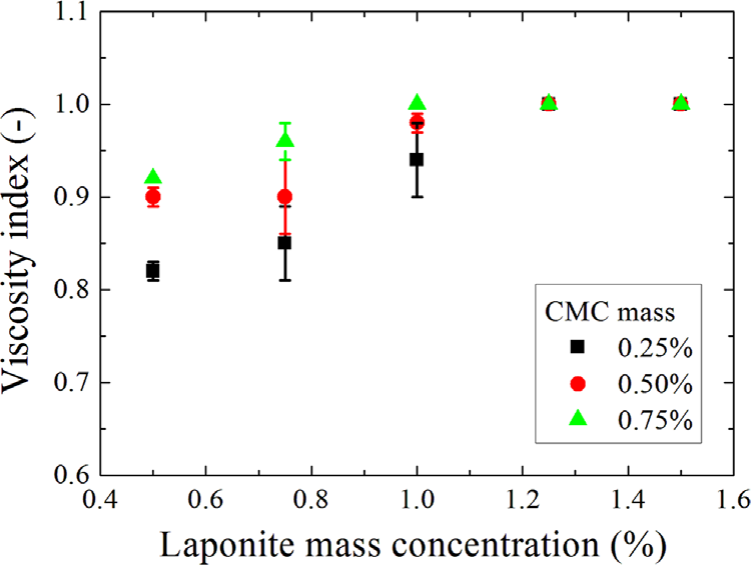
Viscosity indexes as a function of laponite mass concentration for D+20.

**Fig. 11:**
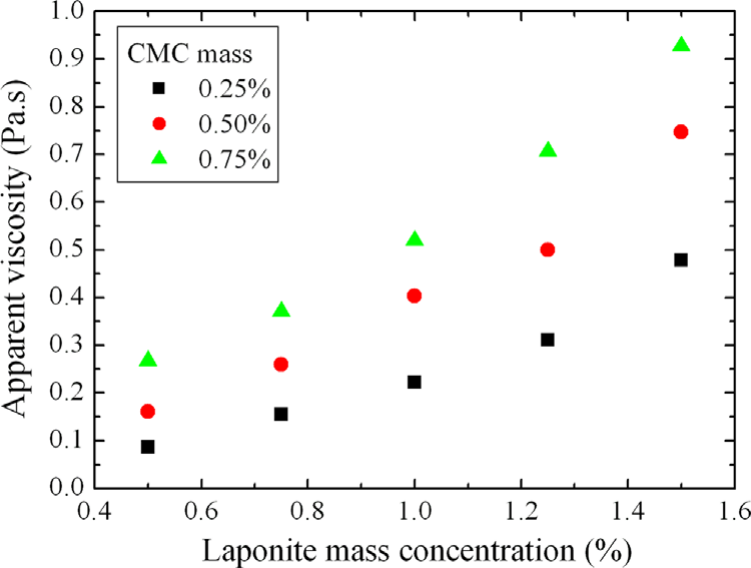
Apparent viscosity for $\overset{˙}{\gamma }=50\;s^{-1}$ as a function of laponite mass concentration for D+20.

**Table 1:** Herschel–Bulkley parameters for 15 samples.

**Sample**	**Yield stress (Pa)**						**Viscosity index (-)**						**Consistency (Pa s^n^)**					
	***D+1***		***D+7***		***D+20***		***D+1***		***D+7***		***D+20***		***D+1***		***D+7***		***D+20***	
	Avg	Std dev	Avg	std dev	Avg	Std dev	Avg	Std dev	Avg	Std dev	Avg	Std dev	Avg	Std dev	Avg	Std dev	Avg	Std dev
**Laponite 0.50%+CMC 0.25%**	1.6	0.2	1.5	0.2	1.5	0.2	0.87	0.02	0.87	0.01	0.82	0.01	0.079	0.003	0.083	0.012	0.113	0.010
**Laponite 0.50%+CMC 0.50%**	3.5	0.3	3.4	0.3	3.5	0.6	0.87	0.02	0.89	0.01	0.90	0.01	0.160	0.032	0.137	0.023	0.132	0.016
**Laponite 0.50%+CMC 0.75%**	5.9	0.5	6.1	0.7	6.0	0.6	0.88	0.01	0.87	0.01	0.92	0.00	0.239	0.021	0.254	0.025	0.200	0.023
**Laponite 0.75%+CMC 0.25%**	2.7	0.0	2.9	0.2	3.6	0.4	0.85	0.02	0.84	0.04	0.85	0.04	0.141	0.023	0.162	0.042	0.149	0.050
**Laponite 0.75%+CMC 0.50%**	5.5	0.2	5.3	0.5	6.1	0.3	0.88	0.02	0.89	0.02	0.90	0.04	0.200	0.035	0.178	0.032	0.200	0.054
**Laponite 0.75%+CMC 0.75%**	9.2	0.4	9.8	0.7	9.5	0.3	0.97	0.01	0.94	0.00	0.96	0.02	0.217	0.022	0.256	0.025	0.210	0.024
**Laponite 1.0%+CMC 0.25%**	4.6	0.3	5.8	0.5	6.3	0.4	0.88	0.02	0.89	0.01	0.94	0.04	0.152	0.023	0.166	0.017	0.121	0.034
**Laponite 1.0%+CMC 0.50%**	7.8	0.3	8.7	0.2	9.8	0.3	0.96	0.01	0.97	0.01	0.98	0.01	0.221	0.029	0.243	0.030	0.229	0.025
**Laponite 1.0%+CMC 0.75%**	13.7	0.5	14.4	0.3	16.3	1.1	0.96	0.01	0.97	0.01	1.00	0.00	0.288	0.022	0.288	0.020	0.193	0.022
**Laponite 1.25%+CMC 0.25%**	7.8	0.4	10.0	0.9	11.1	1.2	0.93	0.02	1.00	0.00	1.00	0.00	0.155	0.010	0.112	0.007	0.090	0.010
**Laponite 1.25%+CMC 0.50%**	12.9	1.3	13.9	0.3	16.5	1.2	0.99	0.01	1.00	0.00	1.00	0.00	0.170	0.045	0.154	0.008	0.170	0.011
**Laponite 1.25%+CMC 0.75%**	20.3	1.4	22.2	0.8	25.1	2.0	1.00	0.00	1.00	0.00	1.00	0.00	0.224	0.043	0.226	0.024	0.205	0.021
**Laponite 1.50%+CMC 0.25%**	12.9	0.2	16.1	0.5	18.9	1.6	1.00	0.00	1.00	0.00	1.00	0.00	0.131	0.014	0.123	0.001	0.101	0.037
**Laponite 1.50%+CMC 0.50%**	20.8	0.3	22.7	0.8	26.0	0.3	1.00	0.00	1.00	0.00	1.00	0.00	0.251	0.028	0.244	0.025	0.228	0.028
**Laponite 1.50%+CMC 0.75%**	30.0	0.5	34.2	0.6	36.6	0.9	1.00	0.00	1.00	0.00	1.00	0.00	0.270	0.031	0.261	0.026	0.196	0.021
